# Knotted double j ureteral stent: a case report and literature review

**DOI:** 10.11604/pamj.2022.43.5.34538

**Published:** 2022-09-05

**Authors:** Omar Jendouzi, Aziz Lamghari, Mounir Jamali, Amine Harchaoui, Mohamed Alami, Ahmed Ameur

**Affiliations:** 1 Department of Urology, Military Teaching Hospital, Rabat, Morocco

**Keywords:** Knot ureteral stent, encrustation, urological complication, case report

## Abstract

Many complications due to double j (DJ) stent placement have been reported. DJ stent knotting is a rare complication, with only a few cases reported in the literature. We presented a case of DJ stent knotting and reviewed the literature regarding this complication. We reported a 20-year-old man with a history of cystinuria and ureteral stone managed with retrograde ureteroscopy and holmium laser three months ago. The patient comes for DJ stent removal. Firstly, we tried to remove the DJ stent via the cystoscopic procedure, which failed. A fluoroscopic image revealed a knotted DJ stent lodged at the ureteropelvic junction and was removed via holmium laser ureteroscopic procedure without complications. In conclusion, when cystoscopic procedure with simple traction fails to remove DJ stents, multimodality urological procedures such as holmium laser should be tried, especially in patients with urolithiasis predisposing factors.

## Introduction

The use of indwelling ureteral double j (DJ) stent has become an integral part of the contemporary urological practice since its introduction in 1978. However, DJ stent placement may lead to complications, including stent migration, stent incrustation, and discomfort [1,2].

The knotting of the proximal ureteric DJ stent is an extremely rare condition in endourology, and the removal of a knotted DJ stent can be challenging with various techniques reported, from simple traction to open surgery [2]. We presented a case of knotting proximal DJ stent and reviewed the literature concerning this unusual complication.

## Patient and observation

**Patient information:** a 20-year-old male patient presented to our department to undergo an elective removal of a DJ ureteral stent ( 7 Fr, 26 cm, coloplast) inserted three months ago during a ureteroscopy for ureteral stones. The patient had a previous history of cystinuria under medical treatment (alkalinizing agents) and dietary measures. Several sessions of extracorporeal lithotripsy, flexible and rigid ureteroscopies were performed for recurrent kidney stones. The patient had no family history of cystinuria.

**Clinical findings:** no bothersome symptoms were reported, and the physical examination was strictly normal.

**Figure 1:**
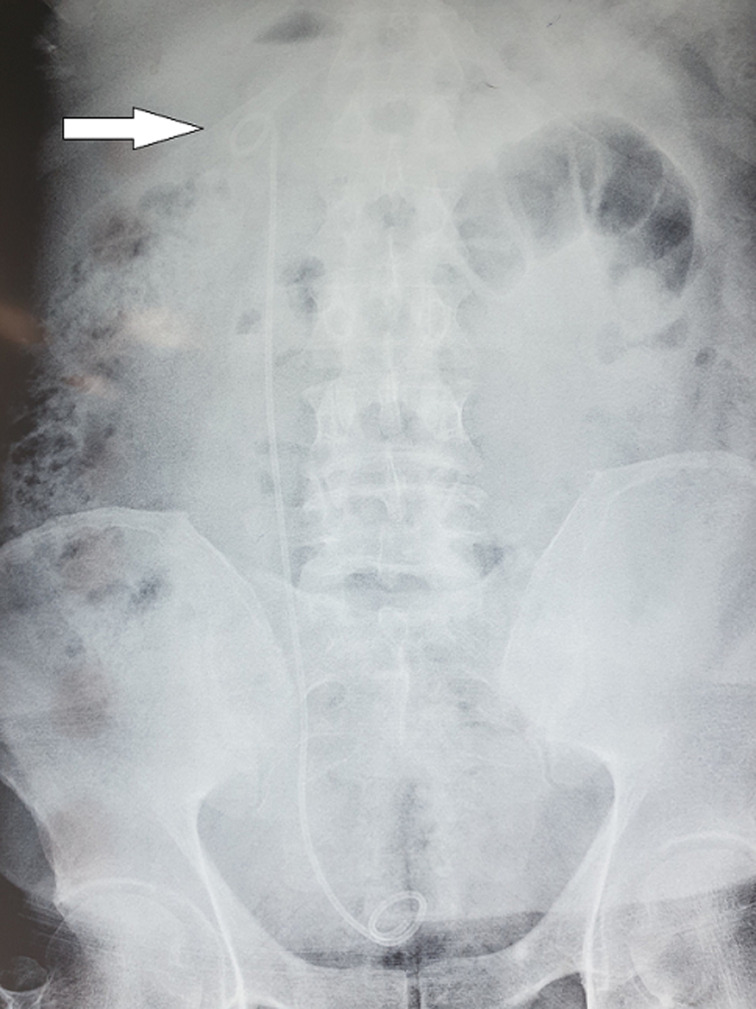
plain radiography X-ray before stent removal: no knotting in the stent (arrow)

**Diagnostic assessment:** blood tests revealed: blood urea nitrogen: 35 mg/dl, and creatinine: 1.3 mg/dl. Urine analysis showed microscopic hematuria (15-20 RBCs/HPF) and many pass cells (20 WBCs/HPF). A plain radiograph X-ray of the abdomen showed no abnormalities in the stent (Figure 1).

**Figure 2:**
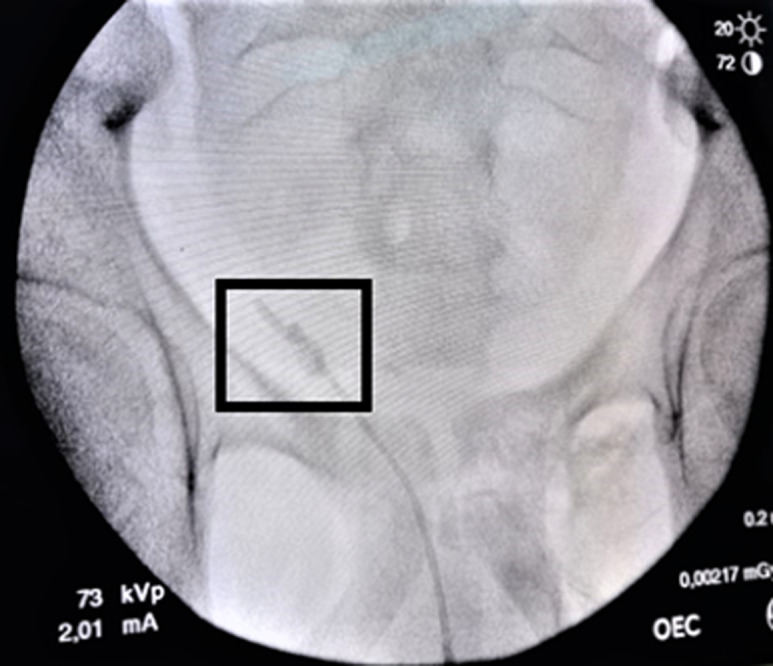
appearance of the knotted double-j stent on the fluoroscopic image (square)

**Figure 3:**
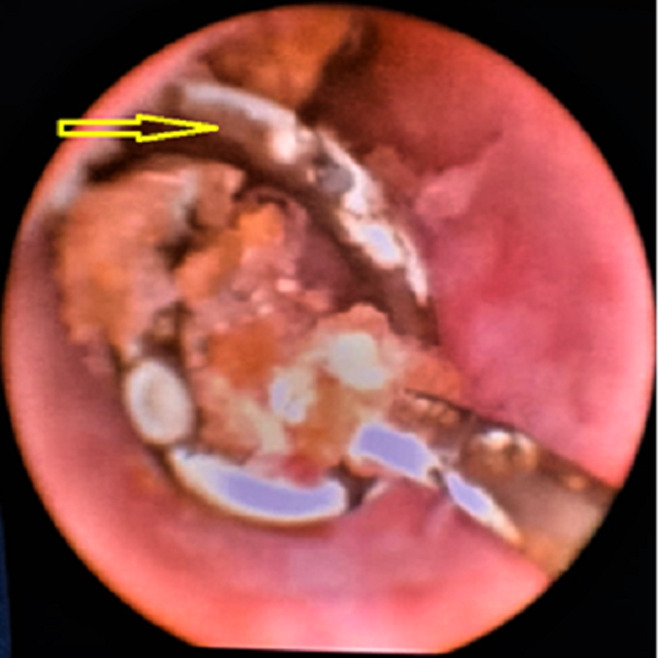
endoscopic view of the proximal end of the stent knotted and encrusted in the pelvic ureter (arrow)

**Figure 4:**
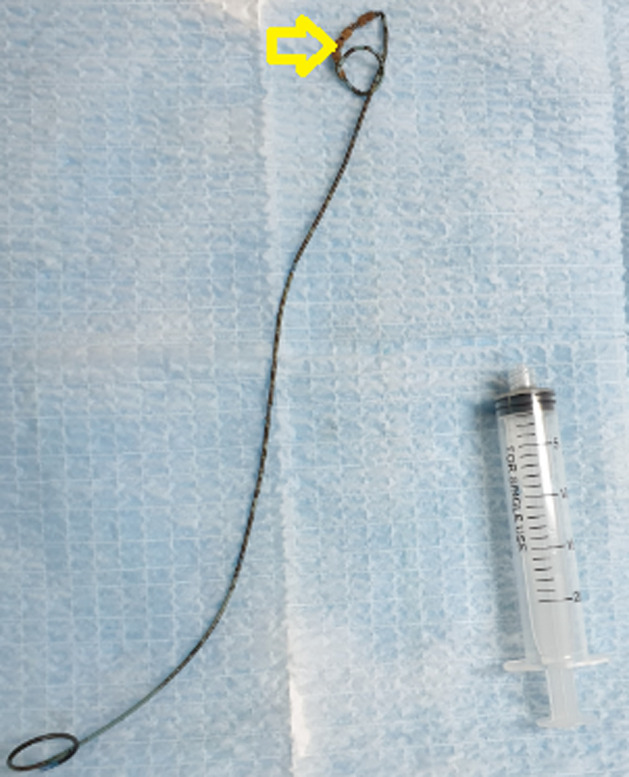
ureteral DJ stent after removal with knotted part (arrow)

**Therapeutic interventions:** we first attempted to remove the DJ ureteral stent via cystoscopic procedure with gentle traction under local anesthesia, but we noted a resistance. So, the decision was to stop the maneuver and transfer the patient to the operative room. Under general anesthesia and fluoroscopic guidance, a fluoroscopic image revealed a knotted stent lodged at the ureteropelvic junction (Figure 2). Firstly, a hydrophilic guidewire was inserted in the ureteral catheter past the knotted stent and coiled in the renal pelvis for safety. Then, a 6-French ureteroscope was advanced into the ureter, and a knot was seen in the proximal end of the DJ stent (Figure 3). Finally, a 2100 nm holmium laser was performed with a setting of 10 Hz and 0.6 J. The stone was broken, and the knot untied. Then, the ureteric DJ stent was entirely removed by grasping forceps (Figure 4). Retrograde urethrography at the end of the procedure excluded any ureteral injury, and a 7-F ureteral catheter was inserted on the guidewire until it was in the renal pelvis. On the third postoperative day, the ureteral catheter was removed.

**Follow-up and outcome:** the postoperative period was uneventful, without complications. After three postoperative days, the patient was discharged with regular follow-up.

**Patient perspective:** the patient was happy with the successful outcome of the surgery.

**Informed consent:** a written informed consent was obtained from the patient for participation in our study.

## Discussion

Indwelling ureteral DJ stent complications include irritative voiding symptoms, flank and suprapubic pain, vesicorenal reflux, stent malposition, hematuria, urinary tract infection, fever, stent encrustation, inadequate obstruction relief, stent migration, stent rupture, ureteral perforation, erosion, and fistulization [3]. Stent knotting is an extremely rare complication. To our best knowledge, only a few cases have been identified in the literature since Groeneveld *et al*. first reported it in 1989 [2,4]. Table 1 summarized the previously recent published cases regarding DJ stent knotting [5-11]. Knotting at the proximal end of the DJ stent was noticed in the vast majority of reported cases (90%) [12]. Most of the patients had no symptoms. Unexpected resistance during the removal of the DJ stent was the typical presentation, and knotting could be detected by plain radiography as described in our report [2].

**Table 1:** reported published cases of knotted ureteral DJ stents in literature

Study/year	Indication	Stent size	Knot location	Knotted stent removal
Bhirud/2012	Post PCNL	NA	Mid-stent	Percutaneous removal
Ahmadi *et al*./2015 (4 cases)	Obstructive uropathy (2 cases) and staged procedure for ureter calculus (2 cases)	6 - 7 Fr	Proximal	Holmium laser
Kim *et al*./2015	Renal stone for ESWL	6 Fr	Proximal	Percutaneous removal with folded terumo guidewire
Manohar *et al*./2015 (4 cases)	Obstructive uropathy (3 cases) mid-ureter injury (1 case)	4,8 - 6 Fr	Proximal	Gentle traction (1 case); holmium laser (2 cases); percutaneous removal (1 case)
Bradshaw *et al*./2020	Post-RIRS	NA	Proximal	Gentle traction
Cho *et al*./2020	Post-RIRS	6 Fr	Proximal	Ureteroscopy
Choo ZW *et al*./2021	Obstructive uropathy	6 Fr	Proximal	Holmium laser
Present study/2022	Obstructive uropathy	7 Fr	Proximal	Holmium laser

PCNL: percutaneous nephrolithotomy; ESWL: extracorporeal shock wave lithotripsy; RIRS: retrograde intrarenal surgery

The ureteral stent knot formation may occur during the implantation or removal processes. Many contributing factors to stent knot formation have been reported. According to literature, stent diameters ranging from 4.7 to 7 Fr are associated with DJ stent knotting with equal distribution. So, the small diameter does not seem to predispose to this condition [7]. The presence of lithogenic urine coupled with prolonged indwelling DJ stent times may increase the risk of DJ stent encrustation but not knotting [2]. Our patient had a history of cystinuria and an indwelling DJ stent for more than three months.

Excessive DJ stent length and coil formation have been hypothesized as a possible cause of ureteral stent knot formation in patients with short ureters, such as segmental ureter resection or renal transplantation. Therefore, controlling the length of the stent in the ureter can avoid knot formation [7,10]. Multilength DJ stents are associated with a lower risk of stent migration; but seem to increase the risk of knot formation and renal stones, potentially influencing proximal coil configuration during stent positioning [2,7]. The close follow-up of stented patients is valuable for the early detection of morbidity or complications, especially in patients with an increased risk of stone formation. Ureteric DJ stent monitoring is essential and should include regular monthly urine cultures, monitoring of serum creatinine levels, and plain radiographic X-ray [13].

Since there are no guidelines for managing this challenging condition, various techniques have been reported. Gentle traction has demonstrated its success for DJ stent retrieval in approximately one-third of the reported cases. Although traction could be easily performed, it can worsen the situation and make the knot tighter. If strong resistance is encountered during traction, the risk of severe ureteral trauma or loss of renal function is essential; and alternative intervention should be considered [7,11].

Some urologists used sterile vaseline as an adjunct maneuver, while others used continuous traction for three days by tying the distal coil with a standard catheter strip to the leg and combining it with extracorporeal shock wave lithotripsy (ESWL) to the site of knot encrustation [14,15]. Baldwin *et al*. used an amplatz 0.038 super stiff guide wire through the stent lumen to untie the knot [16]. Flam *et al*. inserted a second ureteral stent alongside the knotted stent to promote dilatation and removed the stent one week later during ureteroscopy using a 5F alligator forceps [17]. The Valsalva effect obtained by forceful coughs has been related by Eisner *et al*. but it certainly will not be helpful in most cases [15].

Retrograde ureteroscopy with holmium laser to fragment the knotted stent is usually the endourological procedure of choice and has been recommended as a first-line option as it is a safe and reproducible way to untie the knot and remove the DJ stent [6]. Similarly, in our case, the successful removal of the knotted stent by holmium laser smashed the encrustation and allowed us to remove the DJ stent completely by grasping forceps. Limitation of its use includes narrow ureter caliber and difficulty accessing the knot. Braslis *et al*. proposed transecting the stent with a laser and pushing the proximal part into the kidney, which can be retrieved by antegrade access [18].

Further invasive interventions should be considered when conservative methods have been unsuccessful. Percutaneous nephrostomy and antegrade retrieval of the stent should be considered only if the retrograde access is difficult or impossible. Bradshaw *et al*. used the antegrade method to remove the DJ stent in a patient with a cystectomy and ileal conduit [7]. Open surgical removal, such as open ureterotomy, is an invasive procedure; among the 30 published cases, only one case has been reported [19].

## Conclusion

Ureteral DJ stent knotting is a rare urological complication. When simple traction with a cystoscopic procedure fails to remove it, multimodality urological procedures such as holmium laser should be tried, especially in patients with predisposing factors for urolithiasis. To prevent this occurrence, monitoring the DJ stent in place is mandatory, especially in lithogenic urine; moreover, the stent should be promptly removed when no longer needed and changed periodically if chronically indwelling.
